# Epidemiological profile of dengue in Champasak and Savannakhet provinces, Lao People's Democratic Republic, 2003–2020

**DOI:** 10.5365/wpsar.2022.13.4.932

**Published:** 2022-11-23

**Authors:** Sumaira Zafar, Hans J Overgaard, Tiengkham Pongvongsa, Nanthasane Vannavong, Sysavanh Phommachanh, Oleg Shipin, Joacim Rocklöv, Richard E Paul, Md Siddikur Rahman, Mayfong Mayxay

**Affiliations:** a Department of Environmental Engineering and Management, Asian Institute of Technology, Bangkok, Thailand.; b Faculty of Science and Technology, Norwegian University of Life Sciences, Ås, Norway.; c Department of Microbiology, Faculty of Medicine, Khon Kaen University, Khon Kaen, Thailand.; d Savannakhet Provincial Health Department, Phonsavangnuea, Lao PDR.; e Champasak Provincial Health Office, Pakse, Lao PDR.; f Institute of Research and Education Development, University of Health Sciences, Ministry of Health, Vientiane, Lao PDR.; g Department of Public Health and Clinical Medicine, Section of Sustainable Health, Umeå University, Umeå, Sweden.; h Unité de la Génétique Fonctionnelle des Maladies Infectieuses, Institut Pasteur, Paris, France.; i Department of Statistics, Begum Rokeya University, Rangpur, Bangladesh.; j Lao-Oxford-Mahosot Hospital-Welcome Trust Research Unit, Microbiology Laboratory, Mahosot Hospital, Vientiane, Lao PDR.; k Centre for Tropical Medicine and Global Health, University of Oxford, Oxford, United Kingdom of Great Britain and Northern Ireland.

Dengue is the most prevalent vector-borne disease in south-eastern Asia. Caused by the four dengue virus serotypes (DENV-1–4) and transmitted by *Aedes* mosquitoes, primarily by *Aedes aegypti*, the prime contributors to the emergence and spread of dengue are favourable climatic conditions, urbanization and international trade and travel. (*1*)

Dengue emerged as a public health concern in Lao People's Democratic Republic (Lao People's Democratic Republic PDR) in 1983, following its first major outbreak in the capital city of Vientiane, during which 1759 cases of dengue haemorrhagic fever (DHF) were recorded. (*2*) Since then, the country has experienced multiple outbreaks, not just in the capital but also in other parts of the country. About 40% of all dengue cases reported in Lao People's Democratic Republic PDR during 1985–1989 were from Vientiane, with the highest dengue activity occurring during the monsoon season (May to October).

Previous studies of dengue in Lao People's Democratic Republic PDR have focused on a specific province or region and/or have relied on short-term dengue case data. In contrast, this study summarized dengue surveillance data spanning an 18-year period, 2003–2020, from the two most affected southern provinces in Lao People's Democratic Republic PDR. It was designed to inform risk assessment of dengue transmission as well as prevention and control strategies.

## Methods

### Study area

The current study was conducted within a larger project (DENCLIM project; 2018–2021) which aimed to evaluate the effects of environmental change and climatic variability on community vulnerability and exposure to dengue within four geographically similar, but socioeconomically different, neighbouring provinces in southern Lao People's Democratic Republic PDR and north-eastern Thailand.

**Fig. 1:**
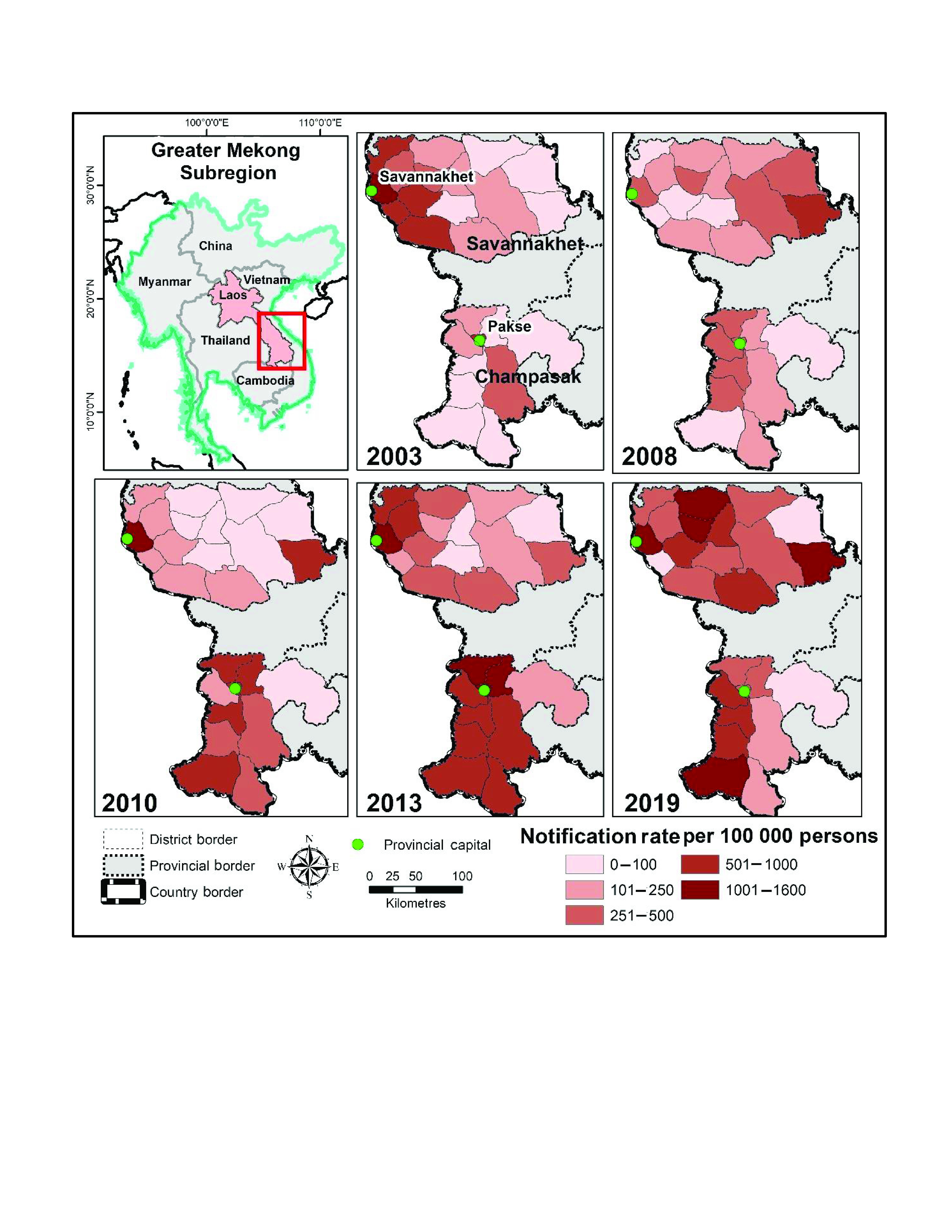
Annual average dengue notification rates in high transmission years by district, Champasak and Savannakhet provinces, Lao PDR, 2003–2020

Lao People's Democratic Republic PDR has three distinct geographical areas (north, central and south). The two most populated provinces in the south, Champasak and Savannakhet, were selected for this study (**Fig. 1**). Champasak and Savannakhet together account for 24% (1.75 million) of the country’s population and both are endemic for dengue with year-round transmission. Peak transmission, however, occurs during the rainy season, from May to October.

### Data collection

Daily reports of dengue cases for Champasak and Savannakhet provinces collected by the two provincial health departments between 2003 and 2020, aggregated at the district level, were used in this study. As per the national dengue surveillance system protocols, all public health practitioners and directors of clinical laboratories must report all dengue cases that meet the dengue case definition within 24 hours of case confirmation to their provincial health department. (*3*) As cases are probably underreported by this surveillance system, data are unlikely to be representative of the true incidence of dengue infection.

Clinically diagnosed dengue cases were initially categorized as either dengue fever (DF), DHF or dengue shock syndrome (DSS). In 2010, Lao People's Democratic Republic PDR adopted the new dengue case classification recommended by the World Health Organization (WHO), (*4*) which categorizes cases as: dengue without warning signs (DWOS), dengue with warning signs (DWS) or severe dengue (SD). (*3*) Dengue cases were recorded in the Champasak province according to the new WHO 2009 classification from 2010 onwards, while Savannakhet only adopted the new classification in 2020. Samples of the notified dengue cases were confirmed by laboratory testing using non-structural protein tests. Data on the prevailing serotypes were obtained from the annual reports of the National Center for Laboratory and Epidemiology and from the provincial health department of Savannakhet.

Population data, based on the 2005 and 2015 censuses, were acquired from the official web portal of the national department of statistics. (*5*) National data on the temporal trends in dengue cases (2003–2020) were also used in the analysis.

### Analysis

#### Dengue notification rate

Available dengue surveillance data included information on the daily number of clinically diagnosed dengue cases and deaths by district, age, sex, occupation, nationality and disease severity. The monthly dengue notification rate was calculated per 100 000 persons (number of cases per month/district population x 100 000). Monthly dengue notification rates were based solely on case data collected by the provincial surveillance system and stored in provincial databases; suspected and unconfirmed cases were not included.

#### Long-term mean of dengue cases

A long-term mean (LTM) method was used to analyse spatiotemporal variations in dengue cases. The LTM was calculated by dividing the total number of dengue cases observed during a specified time period by the total number of time units (i.e. months) in that time period. The time period used in this study was 216 months (2003–2020).

The LTM was used as a threshold to determine the number of months when the monthly number of cases exceeded or remained below the LTM. When the monthly number of cases exceeded the LTM for 3 or more consecutive months, this period was considered to be a “high transmission season.” (*6*) LTMs and the number of months that exceeded them were calculated and mapped for each district within the two provinces.

#### Sociodemographic characteristics of dengue cases

The sociodemographic characteristics of cases including population density, age, sex, occupation and nationality were analysed to identify relative dengue case burdens. The population density of each district in the two provinces was plotted against the dengue notification rate to check for correlation. Dengue cases were also subanalysed by case definition, age group, occupation and nationality to see which groups were most affected.

## Results

### Dengue mortality and notification rates

From 2003 to 2020, 24 479 dengue cases in Champasak and 28 509 in Savannakhet were recorded (**Table 1**). On average, these two provinces combined accounted for 32.6% (Champasak for 17.3%, Savannakhet for 15.3%) of the country’s total number of notified dengue cases (**Table 2**). High transmission seasons occurred in both provinces in 2013 (5387 and 4959 cases in Champasak and Savannakhet, respectively) and again in 2019 (6320 and 3145 cases in Champasak and Savannakhet, respectively). The highest numbers of deaths due to dengue were recorded in 2003 and 2013, followed by 2019 (**Table 2**).

**Table 1:** Characteristics of dengue notifications, Champasak and Savannakhet provinces, Lao People's Democratic Republic PDR, 2003–2020

Characteristic	Champasak		Savannakhet	
	*n*	%	*n*	%
Cases	2003–2020		2003–2020	
Male	12 621	51.6	14 750	51.7
Female	11 858	48.4	13 759	48.3
Total	24 479	100	28 509	100
**Deaths**				
Male	41	46.6	46	46.4
Female	47	53.4	53	53.6
Total	88	100	99	100
**Case definition**				
***Old classification***	**2003–2009**		**2003–2019**	
Dengue fever	7846	97.5	23 716	85.3
Dengue haemorrhagic fever	138	1.7	3406	12.3
Dengue shock syndrome	60	0.7	676	2.4
Total	8044	100	27 798	100
***2009 classification***	**2010–2020**		**2022**	
Dengue without warning signs	13 590	82.7	508	71.4
Dengue with warning signs	2170	13.2	170	23.9
Severe dengue	675	4.1	33	4.6
Total	16 435	100	711	100

**Table 2:** Dengue fever notifications, deaths and notified cases as a proportion of national notifications, Champasak and Savannakhet provinces, Lao People's Democratic Republic PDR, 2003–2020

Year	No. of cases (% of national total)		No. of deaths (Champasak and Savannakhet)	Total no. of cases (Lao People's Democratic Republic PDR)
	Champasak (*n* = 24 479)	Savannakhet (*n* = 28 509)		
2003	914 (5.2)	6315 (35.7)	42	17 690
2004	700 (20.0)	752 (21.4)	13	3507
2005	1487 (27.2)	795 (14.5)	4	5471
2006	1187 (18.7)	314 (4.9)	1	6356
2007	1284 (26.0)	862 (17.4)	0	4943
2008	1557 (37.5)	1935 (46.6)	12	4149
2009	910 (11.8)	177 (2.3)	5	7706
2010	3029 (13.2)	2512 (11.0)	13	22 929
2011	522 (13.5)	50 (1.3)	4	3871
2012	938 (9.4)	225 (2.2)	3	9952
2013	5387 (12.2)	4959 (11.2)	42	44 171
2014	102 (5.9)	15 (0.9)	0	1716
2015	176 (11.0)	34 (2.1)	0	1600
2016	1343 (23.9)	655 (11.7)	13	5617
2017	732 (13.1)	956 (17.1)	5	11 049
2018	1022 (22.2)	922 (20.0)	11	6446
2019	3145 (8.3)	6320 (16.8)	19	37 700
2020	44 (0.5)	711 (8.6)	0	8305

Years with high transmission seasons are shown in bold.

In both provinces, rates of notified dengue cases were higher in the provincial capital districts than in remote districts away from the provincial capitals (**Fig. 1**). The highest annual dengue notification rate was recorded in the south-western districts of Savannakhet in 2019, when rates reached 1595 cases per 100 000 population. Dengue notification rates in both provinces were highly variable and not limited to densely populated areas.

[**insert** Figure 1]

### Spatiotemporal variations in LTMs

**Fig. 2:**
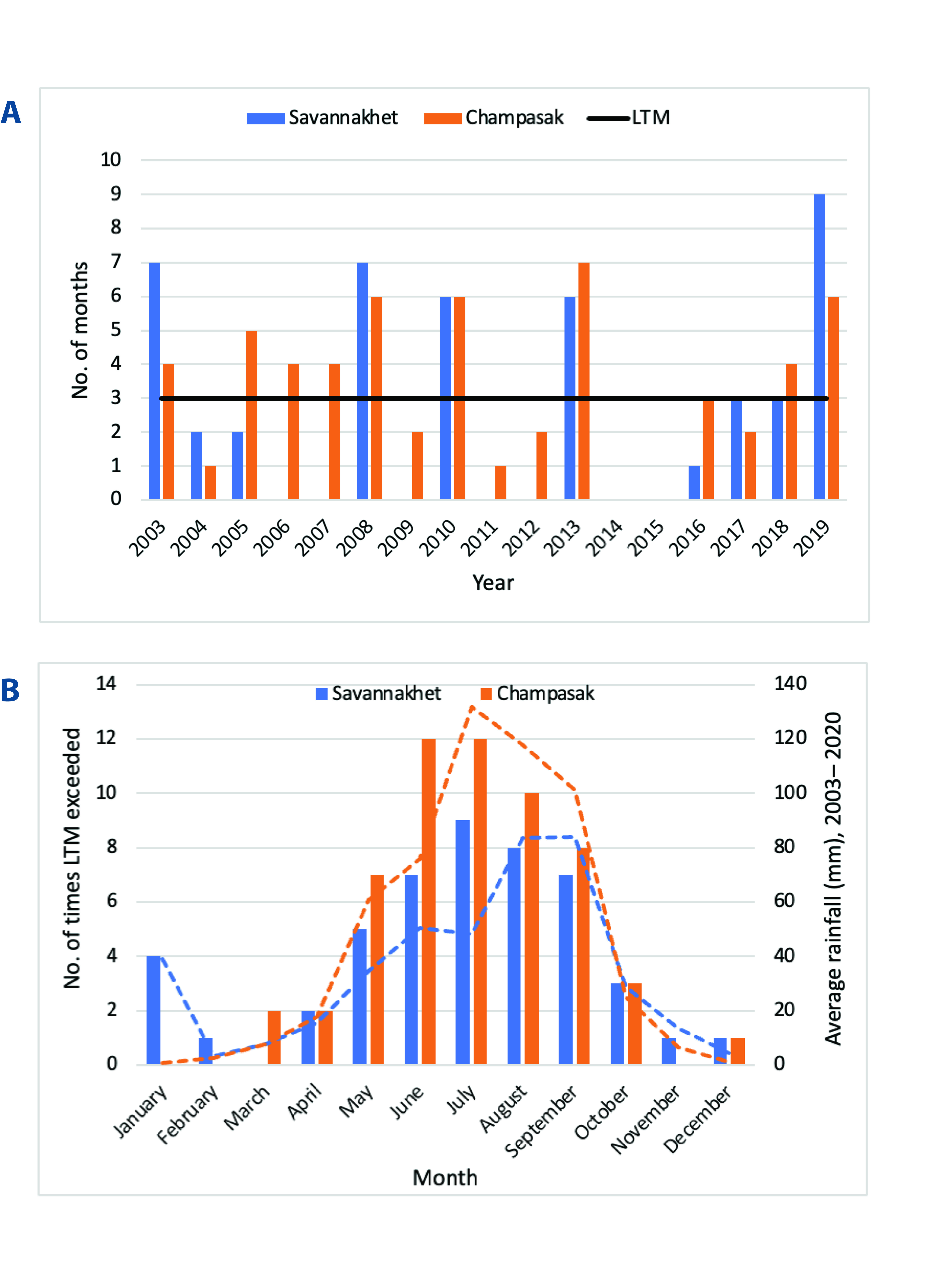
(A) Number of months per year when dengue cases exceeded the long-term mean ; (B) Number of times the long-term mean was exceeded each month compared to average monthly rainfall, Champasak and Savannakhet provinces, Lao PDR, 2003–2020

The LTMs for Champasak and Savannakhet were 113.3 (24 479/216; 95% confidence interval [Cl]: 86.0–140.5) and 132.0 (28 509/216; 95% CI: 92.2–171.7) cases per month, respectively. The number of dengue cases exceeded the LTM for at least 3 consecutive months in 10 of the 18 years of the study period (2003–2020) in Champasak and in 7 of the years in Savannakhet  (**Fig. 2A**). Both provinces experienced extended high-transmission periods. In Champasak, the LTM was exceeded for 7 consecutive months in 2013 (March to September) and for 6 consecutive months in 2008, 2010 and 2019 (March to August). Savannakhet experienced five prolonged epidemic periods, three lasting for 7 months (May to October) in 2003, 2008 and 2013, one for 6 months (June to October) in 2010, and one for 9 months (April to November) in 2019. The number of times the LTM was exceeded was greatest during the rainy season (May to September); during the period of our study, the LTM was most often exceeded in June and July (**Fig. 2B**).

[**insert** Figure 2]

**Fig. 3:**
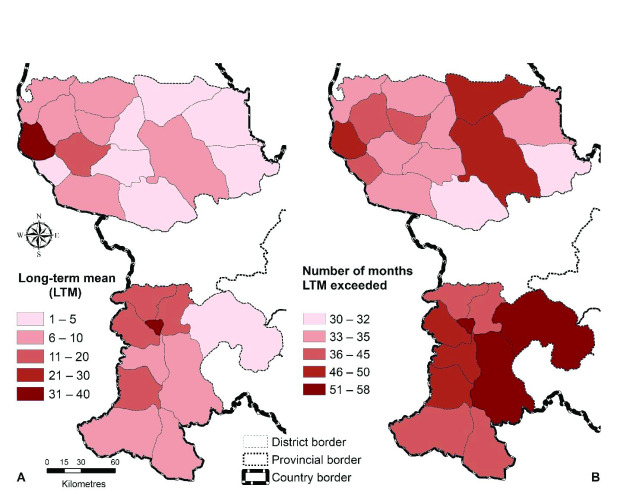
(A) Long-term mean of dengue notifications per month, by district; (B) Number of months when the longterm mean was exceeded, by district, Champasak and Savannakhet provinces, Lao PDR, 2003–2020

In a district-level analysis, the highest LTM values were generally observed in or near the provincial capitals (**Fig. 3A**). In Savannakhet province, three districts exceeded the LTM threshold for 36–45 months and three districts for 46–50 months during the 216-month study period during the 216-month study period. In Champasak province, seven districts exceeded the LTM threshold for 36–45 months and three districts for 56–58 months (**Fig. 3B**).

[**insert** Figure 3]

### Dengue serotypes

Occasional dengue serotype identification conducted by the National Center for Laboratory and Epidemiology showed that in Savannakhet, DENV-1 was detected in 9 of the 11 years between 2003 and 2020 for which serotype data were available. DENV-2 and DENV-4 were also relatively common, being present in 6 out of 11 years, whereas DENV-3 was only found in 2012 and 2013 (**Table 3**). However, DENV-3 was responsible for at least 80% of all reported dengue cases in Lao People's Democratic Republic PDR in 2012 and 2013. Data indicate that in more recent years, DENV-1 and DENV-4 have been the more dominant serotypes, followed by DENV-2, both nationally and in the Champasak and Savannakhet provinces (**Table 3**).

**Table 3:** Prevailing dengue serotypes in Savannakhet and Champasak provinces and Lao People's Democratic Republic PDR, 2003–2020

Year	Serotype		Lao People's Democratic Republic PDR^a^			
	Savannakhet^b^	Champasak	DENV-1 (%)	DENV-2 (%)	DENV-3 (%)	DENV-4 (%)
2003	DENV-1 DENV-2 DENV-4	–	–	–	–	–
2004	–	–	–	–	–	–
2005	DENV-1	–	–	–	–	–
2006	–	–	–	–	–	–
2007	DENV-1 DENV-4	–	–	–	–	–
2008	–	–	–	–	–	–
2009	DENV-1	–	–	–	–	–
2010	DENV-1 DENV-4	–	**38**	30	22	10
2011	–	–	**75**	12	13	0
2012	DENV-2 DENV-3	–	11	9	**80**	0
2013	DENV-1 DENV-2 DENV-3	DENV-2 DENV-3 (*7*)	3	10	**87**	3
2014	–	–	16	17	17	**50**
2015	–	–	**82**	1	1	16
2016	DENV-4	–	11	2	3	**83**
2017	–	–	21	10	< 1	**69**
2018	DENV-1 DENV-2 DENV-4	DENV-1 DENV-2 DENV-4 (*8*)	–	–	–	–
2019	DENV-1 DENV-2	DENV-1 (*9*)	–	–	–	–
2020	DENV-1 DENV-2 DENV-4	–	–	–	–	–

^a^ Country-level serotype data are taken from the annual report of the National Center for Laboratory and Epidemiology for 2017 (unpublished). The prevailing serotype is shown in bold.

^b^ Data are provided by the Savannakhet Health Department.

### Dengue notifications by sociodemographic characteristics

#### Population density

**Fig. 4:**
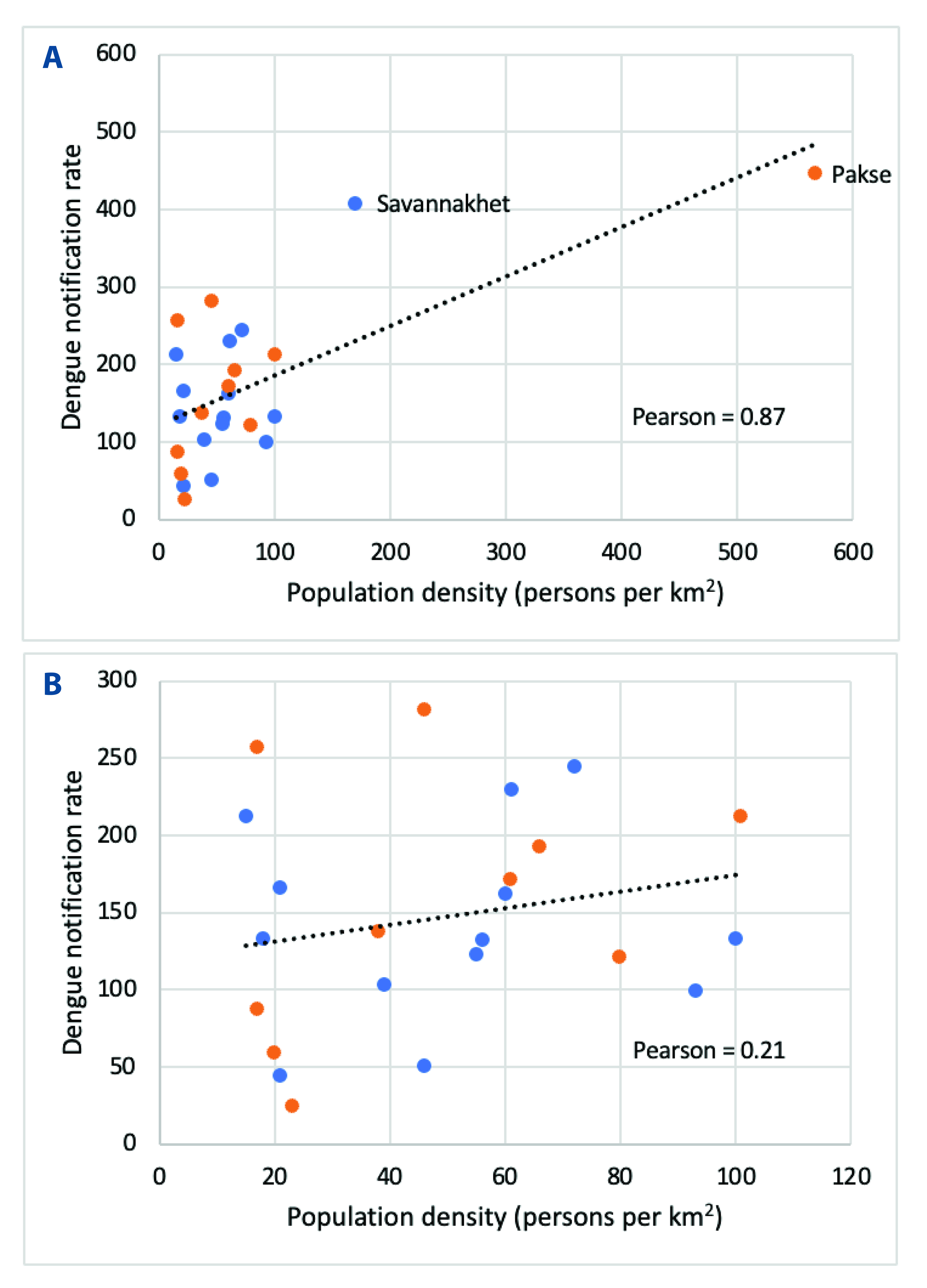
Correlation between population density and average annual dengue notification rate (per 100 000 population) in districts of Champasak (orange points) and Savannakhet (blue points) provinces, (A) including and (B) excluding provincial capitals, Lao PDR, 2003–2020

In both provinces, the highest numbers of dengue notifications were generally observed in the more densely populated provincial capitals and their neighbouring districts (**Fig. 4A**). However, when the capitals were removed, the association between dengue notification rates and population density was not statistically significant (Pearson coefficient = 0.21, *P* = 0.013) (**Fig. 4B**).

[**insert** Figure 4]

#### Age and sex

**Fig. 5:**
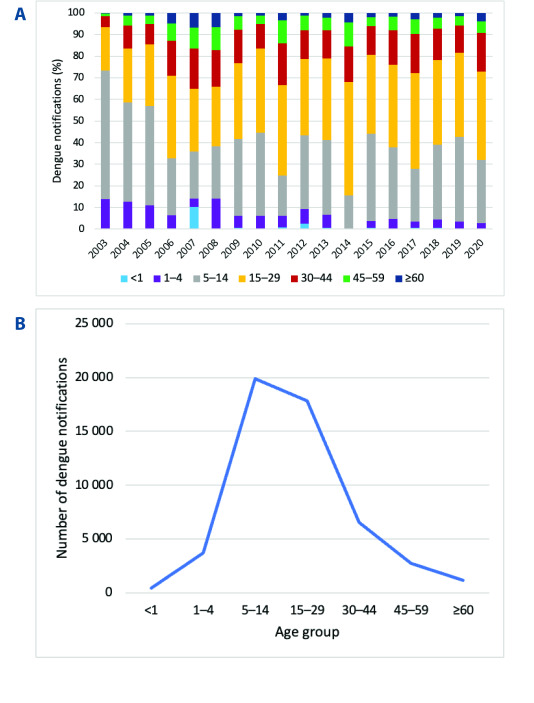
(A) Total number of dengue notifications by age group; (B) Distribution of dengue notifications by age group and year, Lao PDR, 2003–2020

The 5–14-year age group accounted for the highest proportion of cases, followed by the 15–30-year age group. 2007 and 2012 were notable for a higher-than-usual proportion of dengue notifications in those aged < 1 year (**Fig. 5A**).

[**insert** Figure 5]

In all age groups, the majority of dengue infections were categorized as DF (or DWOS). The more severe cases, those categorized as DHF/DWS and DSS/SD, occurred most frequently in those aged 5–14 years old (**Table 4**). Overall, cases were more common in males than females (52% vs 48%) (**Table 1** and **Table 5**); this male excess was also apparent in most age groups, in particular, in the 15–30-year age group. However, in absolute terms, the highest number of deaths occurred in females, with high case fatality rates recorded in those aged under 15 years in both sexes (Table 5).

**Table 4:** Dengue notifications by case definition and age group, Champasak and Savannakhet provinces, Lao People's Democratic Republic PDR, 2003–2020 (percentage of total)

Dengue case definition (old classification/2009 classification)	Age group (years)						
	< 1	1–4	5–14	15–29	30–44	45–59	^3^60
Dengue fever/dengue without warning signs	93.6	87.3	79.0	90.1	92.7	94.8	95.5
Dengue haemorrhagic fever/dengue with warning signs	5.7	10.4	15.7	8.7	6.7	5.0	4.0
Dengue shock syndrome /severe dengue	0.6	2.3	5.3	1.1	0.7	0.2	0.5

**Table 5:** Dengue cases and deaths by age group and sex, Champasak and Savannakhet provinces, Lao People's Democratic Republic PDR, 2003–2020

-	Dengue cases, *n*(%)		Dengue deaths, *n*(%)	
Age group (years)	Female	Male	Female	Male
< 1	112 (0.2)	154 (0.3)	1 (0.89)	0 (0)
1–4	1751 (3.4)	1836 (3.6)	17 (0.97)	16 (0.87)
5–14	9726 (18.8)	10 072 (19.5)	67 (0.69)	57 (0.57)
15–29	8120 (15.7)	9528 (18.4)	11 (0.14)	12 (0.13)
30–44	3214 (6.2)	3294 (6.4)	4 (0.12)	2 (0.06)
45–59	1453 (2.8)	1304 (2.5)	1 (0.07)	0 (0)
^3^60	632 (1.2)	479 (0.9)	1 (0.16)	0 (0)

#### Occupation

**Fig. 6:**
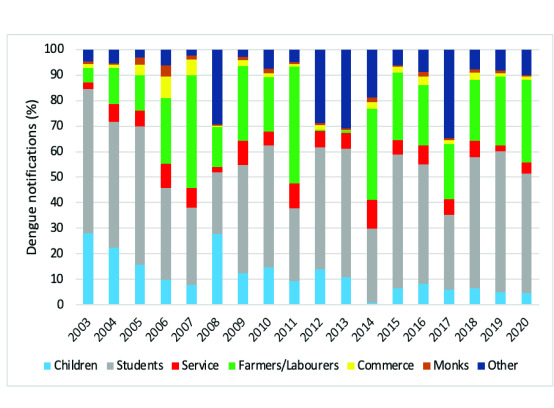
(Proportion of dengue notifications by occupation and year, Champasak and Savannakhet provinces, Lao PDR, 2003–2020

Across the study period, young children (< 5 years), students (5–18 years) and farmers have consistently experienced the greatest burden of dengue; on average, students accounted for 43% of dengue notifications and farmers for a further 22% (**Fig. 6**). However, there has been a shift in the distribution of cases by occupation; whereas the proportion of cases reported in young children and students has fallen (from 84% in 2003 to 60% in 2019), the proportion of dengue notifications in farmers has increased over the same time period (from 6% to 30%). Dengue cases were especially high among farmers in 2007 and 2011, when this group accounted for 44% and 45% of all cases, respectively (**Fig. 6**).

[**insert** Figure 6]

#### Nationality

A total of 218 cases of dengue were recorded among foreign nationals residing in Lao People's Democratic Republic PDR. Of these, the highest numbers were seen in Chinese and Vietnamese citizens, primarily in those engaged in education, rice farming and trading activities (**Table 6**).

**Table 6:** Number of dengue notifications among foreign nationals (*n* = 218), by nationality and occupation, Champasak and Savannakhet provinces, Lao People's Democratic Republic PDR, 2003–2020

Nationality	Occupation							
	All	Children	Students	Service	Farmers	Commerce	Monks	Other
All	218	12	21	3	40	110	3	29
Vietnamese	117	8	16	2	28	36	3	24
Chinese	97	3	5	0	10	74	0	5
Other	4	1	0	1	2	0	0	0

## Discussion

This study describes the long-term dengue epidemic profile for Champasak and Savannakhet, two provinces in southern Lao People's Democratic Republic PDR. Surveillance data from the two provinces indicate a high burden of disease. Moreover, especially high transmission seasons were observed in 2003, 2008, 2010, 2013 and 2019 in both of these two southern provinces, and across the country.

Over the study period, there has been a shift in the geographical distribution of cases in these two provinces. Dengue notification rates were higher in more districts during the 2013 and 2019 high transmission seasons compared with 2003, when dengue notifications were largely confined to the more densely populated districts of western Savannakhet and northern Champasak and the provincial capitals. In 2019, four districts in Savannakhet experienced notification rates in excess of 500 cases per 100 000 population, the highest recorded since the start of the study period in 2003. A similar pattern of increased emergence in new localities has also been reported by neighbouring countries. (*10*) Champasak and Savannakhet provinces are among the four most populated provinces in Lao People's Democratic Republic PDR and have been experiencing extensive development due to agricultural intensification, river dam construction in forests and associated resettlement of workers and inhabitants in remote areas. (*11*) Previous work has also shown a correlation between high density of built-up areas and high levels of development and dengue vulnerability within Champasak and Savannakhet provinces during 2003–2019. (*6*)

The LTM method proved useful for identifying not only the length of dengue epidemics in each year, but also the months with the highest dengue activity and the most affected districts. While the LTM remained high in eastern Savannakhet and northern Champasak throughout the study period, districts in central and western Savannakhet exceeded their LTMs for more months of the year than the eastern districts. This signals a change in dengue case distributions that may be linked to climatic and land cover changes, specifically an increase in mean temperature and in the number of new settlements in previously remote, less developed areas. (*6*)

Dengue notification rates in both provinces tracked the rainy season, with the highest occurrence in June and July. The LTMs followed a similar pattern – higher monthly LTMs were typically observed for at least  3 consecutive months between May and October of each year. These seasonal and spatial patterns in dengue transmission were consistent with those reported in neighbouring south-eastern Asian countries.

Lao People's Democratic Republic PDR has been described as a hyperendemic DENV country, and since the first outbreak in the country in 1979 (followed by the first major outbreak in 1983), all four serotypes have been co-circulating. (*7*-*9*) However, DENV-1 and DENV-2 have consistently been present throughout much of the study period, both across the country as a whole and in the two southern provinces in this study, while the occurrence of DENV-3 and DENV-4 has been more  sporadic. Recent data from the Lao People's Democratic Republic PDR arbovirus surveillance network suggest that since 2016, there has  been a steady decrease in the proportion of cases due to DENV-4 (from 70% to 4% in 2020) and an increase in those caused by DENV-2 (from 7% to 74% in 2020). (*8*)

Population density has been identified as an important driving factor for high dengue transmission. The highest dengue notification rates by far were observed in the densely populated provincial capitals in both southern provinces. Increasing urbanization and high population densities in cities have been associated with an elevated dengue risk with a high vector-to-host ratio. (*1*)

Dengue infections were disproportionately high among children and adolescents aged < 15 years. However, there were signs that age-specific notification rates are beginning to shift to older age groups, as evidenced by the observed 20–30% increase in the number of cases in older adolescents and adults ((*3*)15 years) since 2005 (**Fig. 5A**). Other south-eastern Asian countries have reported falls in their dengue notification rates among those aged < 15 years. The increase in notification rates in older adults (15–45 years) may be explained by the spread of dengue into areas with lower rates of immunity among the population. Changes in circulating dengue virus serotypes (*12*) may also have led to a rise in secondary infections that are considered important risk factors for severe clinical presentations. (*4*)

Dengue case rates among females and males in all age groups remain broadly similar, although we observed a slightly higher case rate in males aged 15–29 years. Similarly, dengue case data reported through national surveillance systems of other countries in the WHO South-East Asia and Western Pacific regions indicate that adult males aged > 15 years are consistently at higher risk of infection than females. (*13*)

In this study, students and farmers were identified as being at higher risk of dengue infection compared with other occupational subgroups, a finding that is consistent with that of another study from Lao People's Democratic Republic PDR, which also found that farmers were the second most affected occupational group. (*14*) Dengue vectors are most active during the daytime. The primary dengue vector, *Ae. aegypti*, is predominantly found indoors, which may account for increased exposure of children and students given that this group spends much of their day inside their homes or classrooms. Farmers may have greater exposure to the secondary vector, *Ae. albopictus*, which oviposits in tree holes and leaf axile. (*14*)

The data collected by provincial health departments inherently come with a few limitations: these include uncertainty in reporting, misdiagnosis and misreporting of symptomatic dengue, and absence of subclinical and asymptomatic infections. For confirmed dengue infections, the serotypes were rarely identified. Travel-related infections are also common in these provinces, but this information was not included in the data and not easy to trace.

In conclusion, this study has characterized the spatiotemporal trends in dengue transmission in southern Lao People's Democratic Republic PDR. Since passive national surveillance data do not always include serotype and entomological information, it is recommended that detailed seroprevalence studies be conducted to further understand dengue epidemiology in Lao People's Democratic Republic PDR. Such studies performed country-wide could help public health authorities develop improved action plans to implement vector control activities each year before the rainy season. As farmers and students under the age of 30 were the most affected groups, combined efforts by the education, agriculture and health ministries to make these groups more aware of the disease risks are recommended. Interventions could include awareness-raising and educational programmes on effective indoor dengue vector control and preventive measures delivered through seminars and medical camps in villages and educational institutions (primary to university level). These could build on the success of the training in epidemic control aimed at village health volunteers, village heads and community schoolteachers currently provided by the International Federation of Red Cross and Red Crescent Societies, which have helped to increase villagers’ and communities’ health preparedness and response. In addition, community-level initiatives to control the spread of dengue should be encouraged; such initiatives might include reducing use of water storage containers, promoting use of larvicides to prevent mosquito breeding, use of mosquito nets and repellents in homes and in agricultural fields and increasing awareness of the risks posed by the accumulation of waste near households.
